# Hospital Incidence and In‐Hospital Mortality of Surgically and Interventionally Treated Aortic Dissections: Secondary Data Analysis of the Nationwide German Diagnosis‐Related Group Statistics From 2006 to 2014

**DOI:** 10.1161/JAHA.118.011402

**Published:** 2019-04-12

**Authors:** Benedikt Reutersberg, Michael Salvermoser, Matthias Trenner, Sarah Geisbüsch, Alexander Zimmermann, Hans‐Henning Eckstein, Andreas Kuehnl

**Affiliations:** ^1^ Department of Vascular and Endovascular Surgery Munich Aortic Centre Klinikum rechts der Isar Technical University of Munich Munich Germany

**Keywords:** aortic dissection, hospital incidence, in‐hospital mortality, secondary data analysis, type A aortic dissection, type B aortic dissection, Cardiovascular Disease, Epidemiology, Cardiovascular Surgery, Health Services, Mortality/Survival

## Abstract

**Background:**

Population‐based data about the incidence and mortality of patients with aortic dissections (ADs) are sparse. Therefore, the hospital incidence and in‐hospital mortality of patients undergoing open or endovascular surgery for type A ADs (TAADs) and type B ADs (TBADs) in Germany were analyzed on a nationwide basis between 2006 and 2014.

**Methods and Results:**

A secondary data analysis of the nationwide diagnosis‐related group statistics, compiled by the German Federal Statistical Office, was performed for patients who were surgically/interventionally treated for AD (*International Classification of Diseases, Tenth Revision, German Modification* [*ICD‐10‐* *GM*] codes I71.00‐I71.07; n=20 533). By using specific procedure codes, a distinction between TAAD (n=14 911/72.6%) and TBAD (n=5622/27.4%) could be made. The standardized hospital incidence of surgically/interventionally treated AD was 2.7/100 000 per year, comprising 2.0/100 000 per year for TAAD and 0.7/100 000 per year for TBAD. The in‐hospital mortality of TAAD was 19.5%; and of TBAD, 9.3%. Both the incidence and in‐hospital mortality increased over the 9‐year period. The share of endovascularly treated TBAD increased steadily during the same time interval. A multilevel multivariable analysis revealed that, for TAAD, age and comorbidity were significantly associated with a higher mortality risk.

The latter was also true for TBAD. Sex was not significantly associated with mortality. A significant association between higher annual center volume and mortality was found for TAAD, but not for TBAD.

**Conclusions:**

This is the first report on hospital incidence and mortality for surgically/interventionally treated AD on a nationwide basis. Overall, in Germany, hospital incidence and mortality of TAAD and TBAD increased over time. In addition, TAAD is performed more safely in high‐volume centers.

## Abstract 2

See Editorial Svensson

**Clinical Perspective**

**What Is New?**

- To the best of our knowledge, this is the first nationwide analysis on the hospital incidence and in‐hospital mortality of aortic dissections, which shows that the incidence and in‐hospital mortality increased over time.
- The emerging endovascular technique significantly lowered the operative risk for patients with type B aortic dissections.
- In addition, an inverse association between center volume and mortality in the treatment of aortic dissection could be shown.

**What Are the Clinical Implications?**

- The results of the study facilitate overall understanding of the epidemiological characteristics of this rare disease.
- From the system perspective, these data may imply that centralization of surgery for aortic dissections might contribute to lower mortality rates and, therefore, may contribute to health policy discussions on structural changes to the healthcare system, such as centralization of cardiovascular services.

## Introduction

An aortic dissection (AD) is a tearing of the intima with consecutive bleeding into the media of the aortic wall. This creates a membrane that divides the aorta into a true and a false lumen. According to the Stanford classification, ADs are classified as type A (TAAD; entry tear in the region of the ascending aorta) and type B (TBAD; entry tear distal to the left subclavian artery).^1^, ^2^ TAAD is a life‐threatening emergency, which requires immediate cardiac surgical treatment. Most TBADs can be treated conservatively by controlling risk factors, such as hypertension, as well as by close surveillance. Indications for surgery are complications, such as aortic rupture, malperfusion, and secondary expansion with aneurysm formation.^3^ Recently, surgical treatment of uncomplicated TBAD has been discussed to be beneficial in terms of prevention of later complications.^4^

Population‐based data about the incidence and mortality of patients requiring surgery for AD are sparse. Published estimations of incidence vary greatly (between 2.3 and 15/100 000 per year),^5^, ^6^, ^7^, ^8^, ^9^, ^10^, ^11^, ^12^ and the overall in‐hospital mortality varies between 10% and 39%.^8^, ^11^, ^13^, ^14^, ^15^ In addition, the latter results are based mainly on registry data from high‐volume centers (eg, the International Registry of Acute Aortic Dissections)^13^ or are the result of population‐based studies limited to small regions.^5^, ^6^, ^7^, ^8^, ^9^, ^11^, ^12^ To date, the 2 largest studies were both based on an administrative data set that did not cover all treated patients. Thus, the study of Wang et al, which analyzed the data of the National Inpatient Sample database, covers only 20% of patients treated in the United States.^15^ Furthermore, the study conducted by Mody et al was based on the Medicare database, which only includes patients aged >65 years.^10^

In summary, to date, no nationwide analysis including all treated patients within one country has been performed. Therefore, the aim of this analysis was to evaluate the hospital incidence and in‐hospital mortality of patients surgically/interventionally treated for AD in Germany (whole system perspective using electronic health records^16^).

## Methods

### Data Source

Using German hospital episode data, all patients treated for AD (*International Classification of Diseases, Tenth Revision, German Modification* [*ICD‐10‐GM*] codes I71.00‐I71.07) between January 1, 2006, and December 31, 2014, were included.

The applied methods have been described in detail previously.^17^, ^18^, ^19^, ^20^, ^21^, ^22^, ^23^, ^24^ In summary, anonymous data of all patients treated in German hospitals are required by law to be reported to the Institute for the Hospital Remuneration System (“Institut fuer das Entgeltsystem im Krankenhaus”). These data are then transmitted in accordance with §21 Hospital Reimbursement Act (“Krankenhausentgeldgesetz”) to the German Federal Statistical Office. As documentation of every inpatient episode is mandatory, this study can be considered complete on a nationwide basis (excluding military and psychiatric services). The validity of the data is monitored by the medical service (“Medizinischer Dienst der Krankenkassen”) of the statutory health insurance companies, which checks the recorded hospital episodes for correctness as part of the financial auditing process. This secondary data analysis was performed in accordance with the Good Practice of Secondary Data Analysis and Reporting of Studies Conducted Using Observational Routinely Collected Health Data guidelines^25^ adapted to the German healthcare system. Data are stored on German Federal Statistical Office servers and could only be accessed through so‐called *controlled remote data processing* using SAS scripts. Individual patient data were not directly available to the authors because of the data‐protection law.

Because of the sensitive nature of the data collected for this study, requests to access the primary data set from qualified researchers trained in human subject confidentiality protocols may be sent to the German Federal Statistical Office (at https://www.forschungsdatenzentrum.de/de/bedingungen) and can be used for secondary data analyses, according to the conditions defined there.

This study conforms to the Declaration of Helsinki and has been approved by the Ethics Committee of the Medical Faculty of the Technical University of Munich (Munich, Germany; reference No. 21/16S). Informed consent was not required. Fulfilment of data protection regulations was controlled by the Research Data Center. The study was not funded by any external sponsor.

### Case Selection and Definition of TAAD and TBAD

A hospital episode covered the period from admission until discharge of a patient. All cases with a principal or secondary diagnosis of AD (*ICD‐10‐GM* codes I71.00‐I71.07) were included, combined with specific procedure codes (according to the German Procedure Classification system) for the treatment of ADs (Table S1).

As reported in numerous publications, a proper distinction between TAAD and TBAD could be made by use of *ICD* codes for AD in combination with the specific procedure codes.^10^, ^14^, ^26^ If the diagnosis (*ICD‐10*) indicates TBAD but treatment codes were specific for TAAD, the patient was classified as having TAAD. If a case had both types of procedure codes, it was also considered as TAAD.

**Figure 1:**
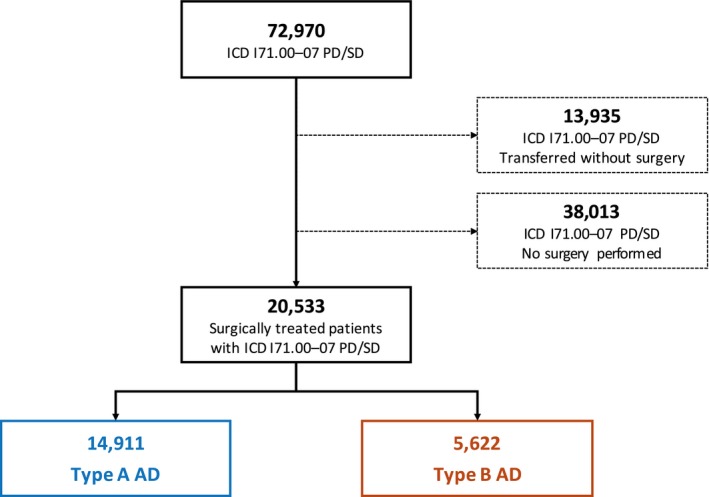
Patient flow diagram. AD indicates aortic dissection; *ICD*,* International Classification of Diseases, Tenth Revision*; PD, principal diagnosis, SD, secondary diagnosis.

Cases with unknown sex and those with unknown or foreign residence were excluded because standardization would otherwise not be possible. Cases with diagnosis codes for AD, but without the above‐mentioned specific procedure codes, were excluded. This was done to avoid double counting (eg, if a patient first received conservative treatment but was then repeatedly admitted to the hospital [resulting in a new administrative case] because of recurrent symptoms and finally underwent surgery [n=38 013; Figure 1]). Because patients cannot be individually identified because of German data protection laws, it was not possible to define whether a single patient had received multiple hospitalizations for surgical treatment of AD. Nevertheless, the number of those cases was considered negligible.

### Case Characteristics and Demographics

Patient data included sex, age, case mix index, type of admission (with referral, without referral, or transferred from another hospital), and type of treatment (for TAAD: open repair; for TBAD: open, endovascular, and hybrid repair). Comorbidities (hypertension, chronic pulmonary disease, diabetes mellitus, chronic kidney disease, cancer, and obesity) were evaluated using codes defined in the Elixhauser Comorbidity Score for primary or secondary diagnosis.^27^, ^28^ Chronic ischemic heart disease, chronic heart failure, cerebrovascular disease, and Marfan syndrome were queried separately as they are not used in the Elixhauser Comorbidity Score (Table S1).

### Primary and Secondary Outcomes

The primary outcomes of this study were hospital incidence and in‐hospital mortality of patients who received operative treatment for TAAD or TBAD. Secondary outcomes included treatment modalities, change in treatment strategies over time, and characterization of perioperative management.

### Statistical Analysis

Categorical variables were reported as absolute numbers and percentages. Continuous, nonnormally distributed variables were shown as the median with first and third quartiles. For comparability with international studies as well as to reflect changes in age and sex, incidence rates were directly standardized using the “European Standard Population 2013.” For trend analysis, the Mann‐Kendall trend test (incidence) and χ^2^ test for trend in proportions (mortality) were used. To test whether age or comorbidity burden (Elixhauser Comorbidity Score) of the patients has changed over the years, the following tests were used: Augmented Dickey–Fuller Test and a difference sign test.^29^ A multilevel logistic regression model with random effects was applied, adjusting for age, sex, comorbidities (modified Elixhauser Comorbidity Score),^27^, ^28^ type of admission (referral, not referred, and transferred from other hospital), type of procedure (endovascular repair and open repair), and clustering of patients in centers enhanced by a temporal first‐order autoregressive covariance structure within a center. For analysis of the association between the number of cases treated per year in a hospital (center volume) and the in‐hospital mortality (volume‐outcome effect), each year hospitals have been grouped by a k‐means clustering algorithm into low‐, medium‐, and high‐volume facilities subject to their annual AD procedures (volume used as an ordinal variable).^30^, ^31^ The clustering algorithm used annual center volume as a separating variable. This was done to avoid arbitrary categorization and to arrange homogeneous clusters based on the data. In addition, a multivariable regression model has been used to analyze the continuous volume‐outcome effect while adjusting for sex, age, comorbidities, and type of treatment (volume used as a metric variable). SAS software, version 9.2 (Microsoft Windows, ©2015; SAS Institute Inc, Cary, NC) was used for controlled remote data processing and statistical analysis. Graphical processing was performed using Microsoft Excel (Redmond, WA) and R, version 3.4.1 (The R Foundation, http://www.r-project.org). A 2‐tailed level of significance of α=0.05 was used for all statistical tests.

## Results

### Patient Characteristics

**Figure 2:**
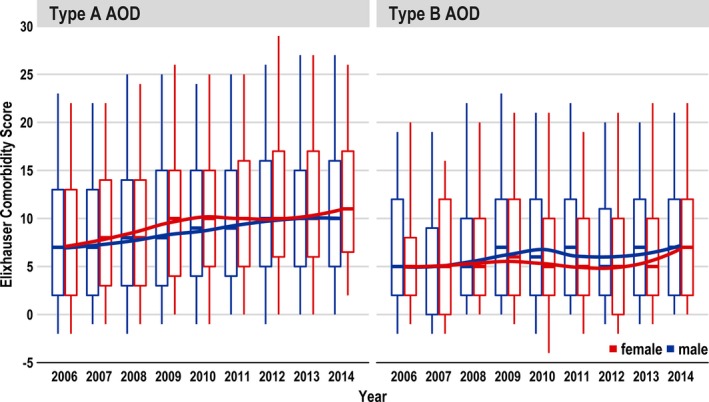
Change of comorbidities represented by the Elixhauser Comorbidity Score of type A and type B aortic dissections (ADs) over the years (2006–2014).

**Figure 3:**
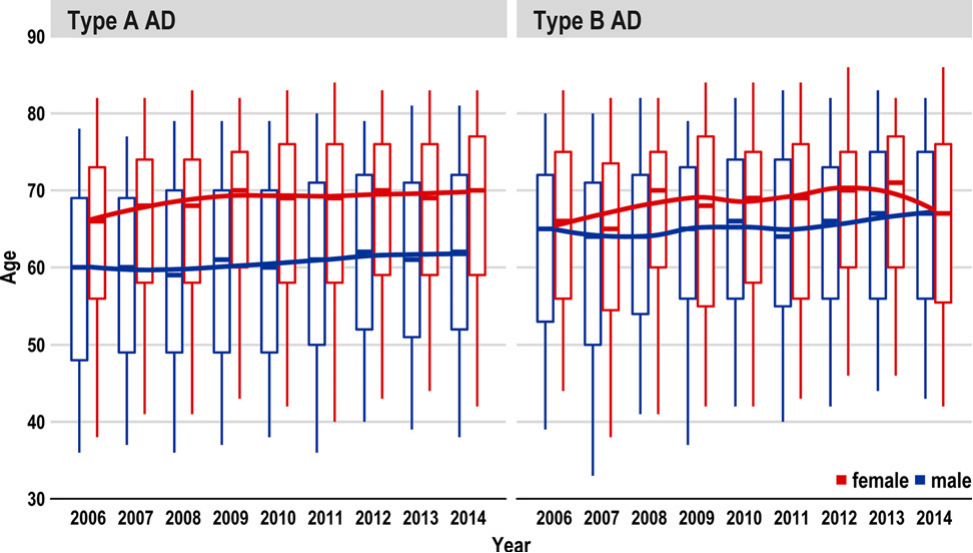
Change of age of type A and type B aortic dissections (ADs) over the years (2006–2014).

Between 2006 and 2014, 20 533 patients (median age, 65 years; quartile 1–quartile 3, 53–73 years) underwent surgery for AD in Germany. Of these patients, 14 911 (72.6%) had TAAD and 5622 (27.4%) had TBAD (Figure 1). Table 1 provides details on patient characteristics. Patients with TAAD were a median age of 64 years (quartile 1–quartile 3, 53–73 years). Patients with TBAD were, on average, 2 years older, and were a median age of 66 years (quartile 1–quartile 3, 56–74 years). The proportion of men was greater in the TBAD than in the TAAD group (71.3% versus 65.0%). Patients with TBAD more often experienced hypertension (77.7% versus 65.3%), chronic kidney disease (20.4% versus 19.3%), and chronic pulmonary disease (12.6% versus 9.1%). On the other hand, chronic ischemic heart disease (22.4% versus 19.9%), chronic heart failure (21.3% versus 10.0%), cerebrovascular disease (18% versus 8.9%), and Marfan syndrome (2.1% versus 1.4%) were more frequent in patients with TAAD. Between 2006 and 2014, age and comorbidity burden increased slightly over time, but there was no significant upwards trend (Figures 2 and 3).

**Table 1:** Characteristics of All Cases Admitted With AD

Characteristics	TAAD	TBAD
No. (%)	14 911 (72.6)	5622 (27.4)
Raw incidence (per 100 000)	2.1 (1.1–2.2)	0.7 (0.6–1.0)
Standardized incidence^a^	2.0 (1.8–2.1)	0.7 (0.6–0.9)
Age, y	64 (53–73)	66 (56–74)
Sex (male), n (%)	9685 (65.0)	4008 (71.3)
Elixhauser Comorbidity Score	9 (4–15)	5 (2–11)
Comorbidities, n (%)		
Chronic ischemic heart disease	3343 (22.4)	1118 (19.9)
Chronic heart failure	3179 (21.3)	564 (10.0)
Cerebrovascular disease	2689 (18)	501 (8.9)
Hypertension	9739 (65.3)	4368 (77.7)
Chronic pulmonary disease	1356 (9.1)	709 (12.6)
Diabetes mellitus	1428 (9.6)	640 (11.4)
Chronic kidney disease	2871 (19.3)	1146 (20.4)
Cancer	150 (1)	135 (2.4)
Obesity	1942 (13)	593 (10.5)
Marfan syndrome	311 (2.1)	79 (1.4)
Type of admission, n (%)		
Admission with referral	3093 (20.7)	2205 (39.2)
Admission without referral	5364 (36)	1997 (35.5)
Transferred from another hospital	6454 (43.3)	1420 (25.3)

If not stated otherwise, continuous data are given as median (first‐third quartile). AD indicates aortic dissection; TAAD, type A AD; TBAD, type B AD.

a Standardized for age and sex using European Standard Population 2013.

### Hospital Incidence

**Figure 4:**
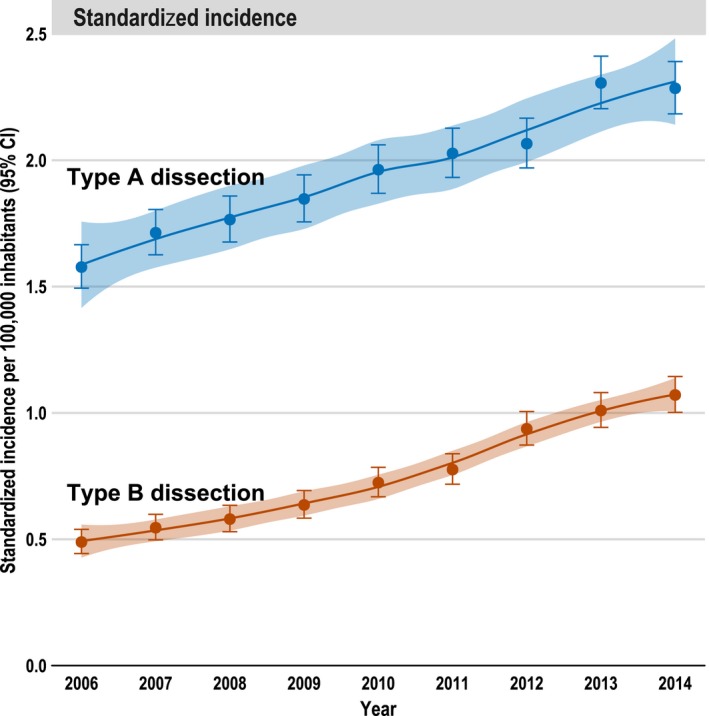
Hospital incidence of type A and type B aortic dissections over the years (2006–2014), standardized per 100 000 inhabitants. Whiskers are 95% CI of incidence/mortality; 95% confidence band refers to LOESS curve.

The median overall hospital incidence of patients treated for any type of AD over the 9‐year period was 2.8/100 000 per year. The incidence of TAAD was 2.1/100 000 per year, and that of TBAD 0.7/100 000 per year. After direct standardization, the incidence for all AD was 2.7/100 000 per year, comprising 2.0/100 000 per year (quartile 1–quartile 3, 1.8–2.0/100 000 per year) for TAAD and 0.7/100 000 per year (quartile 1–quartile 3, 0.6–1.0/100 000 per year) for TBAD (Table 1 and Figure 4).

Over the 9‐year period, the standardized incidence of both TAAD and TBAD increased significantly (*P*<0.001; Mann‐Kendall test). In 2006, the incidences for TAAD and for TBAD were 1.6 and 0.5/100 000 per year, respectively, whereas in 2014, they increased to 2.4 and 1.1/100 000 per year, respectively (Figure 4).

### Treatment

**Figure 5:**
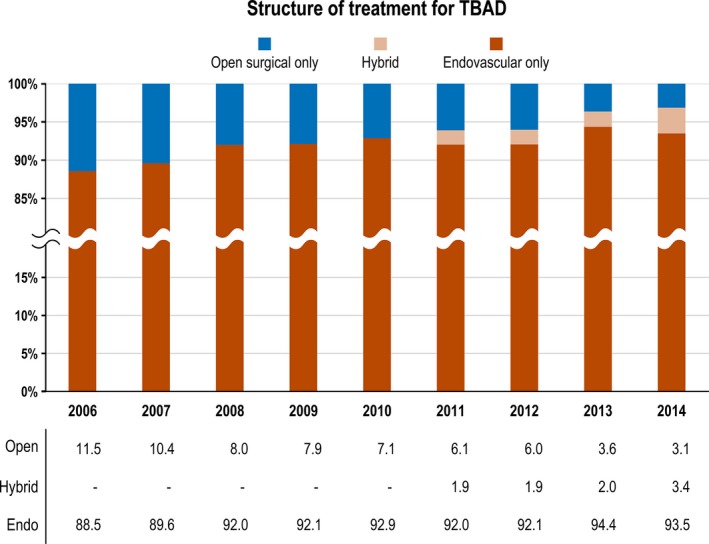
Surgical treatment of patients with type B aortic dissection (TBAD): Proportion of cases treated by open repair, hybrid repair, and endovascular therapy from 2006 to 2014.

Patients with TAAD mainly underwent open repair (94.3%), and in 5.7% of these cases, they received hybrid repair. Patients with TBAD were treated endovascularly in 92.3%, by open repair in 6.4%, and with hybrid repair in 1.3% (Table 2). In TBAD, a constant increase in the proportion of endovascular repair procedures could be observed over the years. When also considering the number of cases treated with a hybrid procedure, the proportion of endovascularly treated patients increased from 88.5% in 2006 to 96.9% in 2014, which was significant within a trend analysis (*P*<0.001) (Figure 5).

**Table 2:** Procedures and Management

Variable	TAAD (n=14 911)	TBAD (n=5622)
Type of surgical treatment		
Open repair	14 054 (94.3)	359 (6.4)
Endovascular	NA	5188 (92.3)
Hybrid repair	857 (5.7)	75 (1.3)
Perioperative management		
Monitoring of evoked potentials	157 (1.1)	81 (1.4)
Spinal catheter use	108 (0.7)	220 (3.9)
Heart‐lung machine use	9916 (66.5)	27 (0.5)
Extracorporeal membrane oxygenation use	433 (2.9)	14 (0.2)
Ventilation	10 454 (70.1)	1569 (27.9)
Duration of ventilation, h	51 (10–175)	0 (0–25)
Length of hospital stay, d	14 (9–22)	13 (8–22)
Cell‐saver auto (re‐)transfusion	3404 (22.8)	247 (4.4)
Cardiopulmonary resuscitation	1591 (10.7)	285 (5.1)
pRBC transfusion		
1–5 pRBCs	4597 (30.8)	1237 (22)
>5 pRBCs	8556 (57.4)	1002 (17.8)
No pRBC transfusion	1758 (11.8)	3383 (60.2)
PC transfusion		
1–5 PCs	9141 (61.3)	392 (7)
>5 PCs	2179 (14.6)	125 (2.2)
No PC transfusion	3591 (24.1)	5105 (90.8)

Data are given as number (percentage) or median (first‐third quartile). NA indicates not applicable; PC, platelet concentrate; pRBC, packed red blood cell; TAAD, type A aortic dissection; TBAD, type B aortic dissection.

The median length of hospital stay was 14 days for surgically/interventionally treated TAAD and 13 days for TBAD. Of patients with TAAD, 69.4% were surgically treated with the assistance of an extracorporeal machine, which provided prolonged cardiac and respiratory support (heart‐lung machine or extracorporeal membrane oxygenation). Prolonged postoperative ventilation was required in 70.1% of cases treated for TAAD and in 27.9% of cases treated for TBAD. In 11.8% of TAAD cases and 60.2% of TBAD cases, there was no documented code that indicates transfusion of red blood cells (Table 2). At least 10.7% of patients with TAAD and 5.1% of patients with TBAD required cardiopulmonary resuscitation. Acute paraplegia was documented in 12% of TAAD cases and 6.7% of TBAD cases. Postoperative dialysis was recorded in almost a quarter (24.6%) of patients with TAAD and in 8.2% of patients with TBAD (Table 3).

**Table 3:** Outcome Overview

Variable	TAAD (N=14 911)	TBAD (N=5622)
In‐hospital mortality overall	2913 (19.5)	522 (9.3)
Perioperative complications		
Acute/recurrent myocardial infarction	541 (3.6)	104 (1.8)
Acute paraplegia/spinal infarction	1787 (12)	375 (6.7)
Stroke	156 (1.0)	16 (0.3)
Acute limb ischemia	368 (2.5)	320 (5.7)
Major limb amputation	17 (0.1)	19 (0.3)
Acute mesenteric infarction	321 (2.2)	170 (3.0)
Acute renal artery infarction	160 (1.1)	118 (2.1)
Dialysis, hemofiltration	3664 (24.6)	462 (8.2)
Bowel resection	146 (1.0)	117 (2.1)
Type of discharge		
Regular discharge	3113 (20.9)	3741 (66.5)
Discharge against medical advice	39 (0.3)	42 (0.7)
Rehabilitation	3818 (25.6)	479 (8.5)
Other hospital	4958 (33.3)	783 (13.9)
Other discharge reason	70 (0.5)	55 (1)

Data are given as number (percentage). TAAD indicates type A aortic dissection; TBAD, type B aortic dissection.

### Morbidity and Mortality

**Figure 6:**
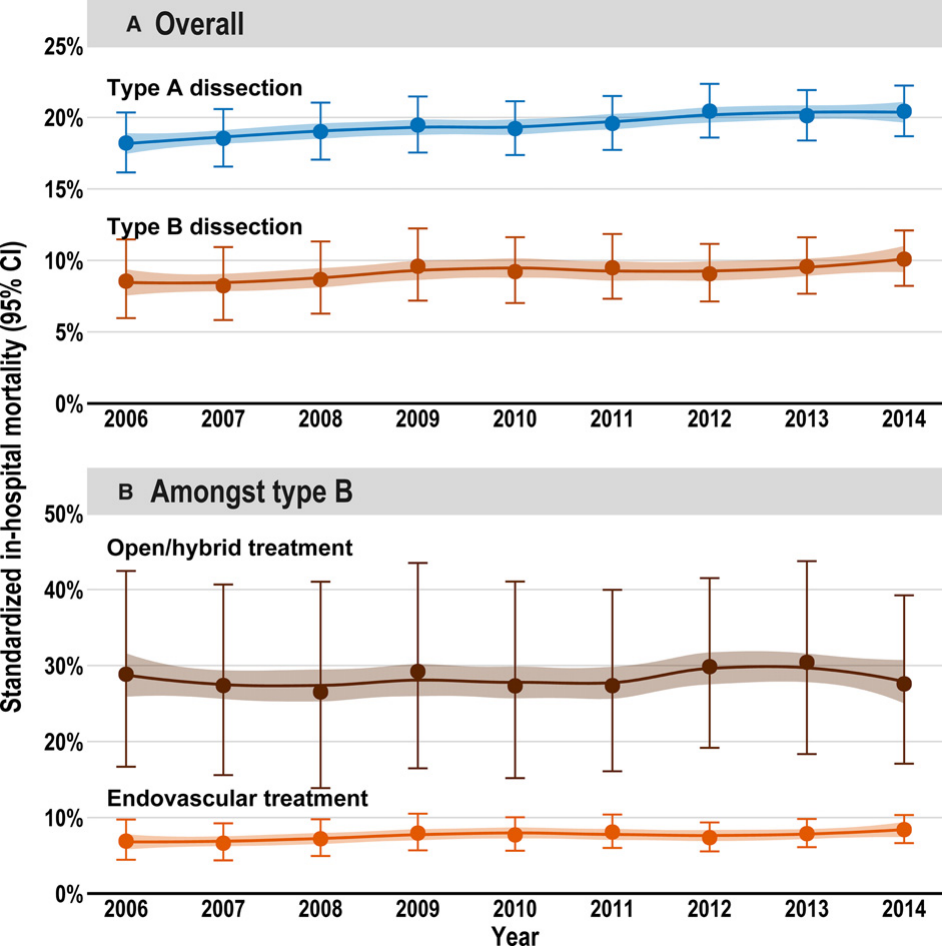
Age‐, sex‐ and risk‐standardized (Elixhauser Comorbidity Score) in‐hospital mortality from 2006 to 2014 for both types of aortic dissection (**A**) and among type B aortic dissections (depending on the received treatment; **B**). Whiskers are 95% CI of mortality; 95% confidence band refers to LOESS curve.

The overall in‐hospital mortality for all types of AD was 16.7%, comprising 19.5% for TAAD and 9.3% for TBAD. In TBAD, the in‐hospital mortality of patients receiving open repair was 34.3%, whereas the figure was 7.6% in patients receiving endovascular repair (Table 3). Characteristics of survivors versus nonsurvivors among those with TAAD and TBAD are given in Tables 4 and 5, respectively. Trend analysis of the age‐ and sex‐standardized mortality showed a significant increase in mortality of TAAD over time (*P*=0.031), whereas no significant trend was apparent in TBAD (TBAD, *P*=0.241; open/hybrid repair, *P*=0.871; endovascular repair, *P*=0.267; Figure 6). Most patients with TAAD (33.3%) were transferred to another hospital after treatment, followed by 25.6% who were discharged to rehabilitation. Most patients with TBAD had a regular discharge home (66.5%; Table 3).

**Table 4:** Survivors Versus Nonsurvivors Among Those With TAAD (n=14 911)

Variable	Survivors (n=11 998)	Nonsurvivors (n=2913)	*P* Value
Age, y	63 (52–72)	68 (57–75)	<0.001
Sex (male)	7848 (65.4)	1837 (63.1)	0.0181
Elixhauser Comorbidity Score	9 (4–15)	10 (5–17)	<0.001
Case mix index	7.2 (5.9–10.9)	8.2 (6.6–12.2)	<0.001
Length of hospital stay, d	15 (11–24)	3 (1–11)	<0.001
Coded comorbidities			
Chronic ischemic heart disease	2463 (20.5)	880 (30.2)	<0.001
Chronic heart failure	2229 (18.6)	950 (32.6)	<0.001
Cerebrovascular disease	2033 (16.9)	656 (22.5)	<0.001
Hypertension	8208 (68.4)	1531 (52.6)	<0.001
Chronic pulmonary disease	1108 (9.2)	248 (8.5)	0.239
Diabetes mellitus	1138 (9.5)	290 (10.0)	0.460
Chronic kidney disease	2307 (19.2)	564 (19.4)	0.891
Cancer	116 (1.0)	34 (1.2)	0.385
Obesity	1570 (13.1)	372 (12.8)	0.673
Marfan syndrome	277 (2.3)	34 (1.2)	<0.001
Type of surgical treatment			
Open repair	11 331 (94.4)	2723 (93.5)	0.050
Hybrid repair	667 (5.6)	190 (6.5)	
Perioperative management			
Monitoring of evoked potentials	129 (1.1)	28 (1.0)	0.660
Spinal catheter use	93 (0.8)	15 (0.5)	0.173
Heart‐lung machine use	7963 (66.4)	1953 (67.0)	0.503
Extracorporeal membrane oxygenation use	84 (0.7)	349 (12.0)	<0.001
Ventilation	8452 (70.4)	2002 (68.7)	0.073
Duration of ventilation, h	79 (32–228)	95 (39–265)	<0.001
pRBC transfusion			
1–5 pRBCs	4144 (34.5)	453 (15.6)	<0.001
>5 pRBCs	6375 (53.1)	2181 (74.9)	
No pRBC transfusion	1479 (12.3)	279 (9.6)	
PC transfusion			
1–5 PCs	7742 (64.5)	1399 (48.0)	<0.001
>5 PCs	1445 (12.0)	734 (25.2)	
No PC transfusion	2811 (23.4)	780 (26.8)	
Cell‐saver auto (re‐)transfusion	2769 (23.1)	635 (21.8)	0.147
Cardiopulmonary resuscitation	670 (5.6)	921 (31.6)	<0.001
Coded perioperative complications			
Acute/recurrent myocardial infarction	290 (2.4)	251 (8.6)	<0.001
Acute paraplegia/spinal infarction	88 (0.7)	4 (0.1)	<0.001
Acute limb ischemia	259 (2.2)	109 (3.7)	<0.001
Major limb amputation	12 (0.1)	5 (0.2)	0.471
Acute mesenteric infarction	119 (1.0)	202 (6.9)	<0.001
Acute renal artery infarction	107 (0.9)	53 (1.8)	<0.001
Dialysis, hemofiltration	2366 (19.7)	1298 (44.6)	<0.001
Bowel resection	72 (0.6)	74 (2.5)	<0.001

Data are given as number (percentage) or median (first‐third quartile). PC indicates platelet concentrate; pRBC, packed red blood cell; TAAD, type A aortic dissection.

**Table 5:** Comparison of Survivors Versus Nonsurvivors Among Those With TBAD (n=5622)

Variable	Survivors (n=5100)	Nonsurvivors (n=522)	*P* Value
Age, y	66 (56–74)	70 (57–77)	0.003
Sex (male)	3640 (71.4)	368 (70.5)	0.712
Elixhauser Comorbidity Score	5 (2–10)	11 (5–18)	<0.001
Case mix index	7.9 (7.0–9.5)	8.6 (7.2–13.1)	<0.001
Length of hospital stay, d	14 (9–22)	7 (3–20)	0.093
Coded comorbidities			
Chronic ischemic heart disease	1024 (20.1)	94 (18.0)	0.284
Chronic heart failure	479 (9.4)	85 (16.3)	<0.001
Cerebrovascular disease	414 (8.1)	87 (16.7)	<0.001
Hypertension	4032 (79.1)	336 (64.4)	<0.001
Chronic pulmonary disease	637 (12.5)	72 (13.8)	0.433
Diabetes mellitus	578 (11.3)	62 (11.9)	0.764
Chronic kidney disease	1021 (20.0)	125 (23.9)	0.039
Cancer	113 (2.2)	22 (4.2)	0.007
Obesity	543 (10.6)	53 (10.2)	0.784
Marfan syndrome	70 (1.4)	9 (1.7)	0.649
Type of surgical treatment			
Open repair	254 (5.0)	105 (20.1)	<0.001
Endovascular	4789 (93.9)	399 (76.4)	
Hybrid repair	57 (1.1)	18 (3.4)	
Perioperative management			
Monitoring of evoked potentials	73 (1.4)	8 (1.5)	1.000
Spinal catheter use	201 (3.9)	19 (3.6)	0.826
Heart‐lung machine use	18 (0.4)	9 (1.7)	<0.001
Extracorporeal membrane oxygenation use	6 (0.1)	235 (45.0)	<0.001
Ventilation	1203 (23.6)	366 (70.1)	<0.001
Duration of ventilation, h	49 (13–223)	110 (29–357)	<0.001
pRBC transfusion			
1–5 pRBCs	1136 (22.3)	101 (19.3)	<0.001
>5 pRBCs	664 (13.0)	338 (64.8)	
No pRBC transfusion	3300 (64.7)	83 (15.9)	
PC transfusion			
1–5 PCs	242 (4.7)	150 (28.7)	<0.001
>5 PCs	66 (1.3)	59 (11.3)	
No PC transfusion	4792 (94.0)	313 (60.0)	
Cell‐saver auto (re‐)transfusion	198 (3.9)	49 (9.4)	<0.001
Cardiopulmonary resuscitation	97 (1.9)	188 (36.0)	<0.001
Coded perioperative complications			
Acute/recurrent myocardial infarction	79 (1.5)	25 (4.8)	<0.001
Acute paraplegia/spinal infarction	55 (1.1)	9 (1.7)	0.268
Acute limb ischemia	254 (5.0)	66 (12.6)	<0.001
Major limb amputation	13 (0.3)	6 (1.1)	0.003
Acute mesenteric infarction	78 (1.5)	92 (17.6)	<0.001
Acute renal artery infarction	94 (1.8)	24 (4.6)	<0.001
Dialysis, hemofiltration	259 (5.1)	203 (38.9)	<0.001
Bowel resection	59 (1.2)	58 (11.1)	<0.001

Data are given as number (percentage) or median (first‐third quartile). PC indicates platelet concentrate; pRBC, packed red blood cell; TBAD, type B aortic dissection.

**Figure 7:**
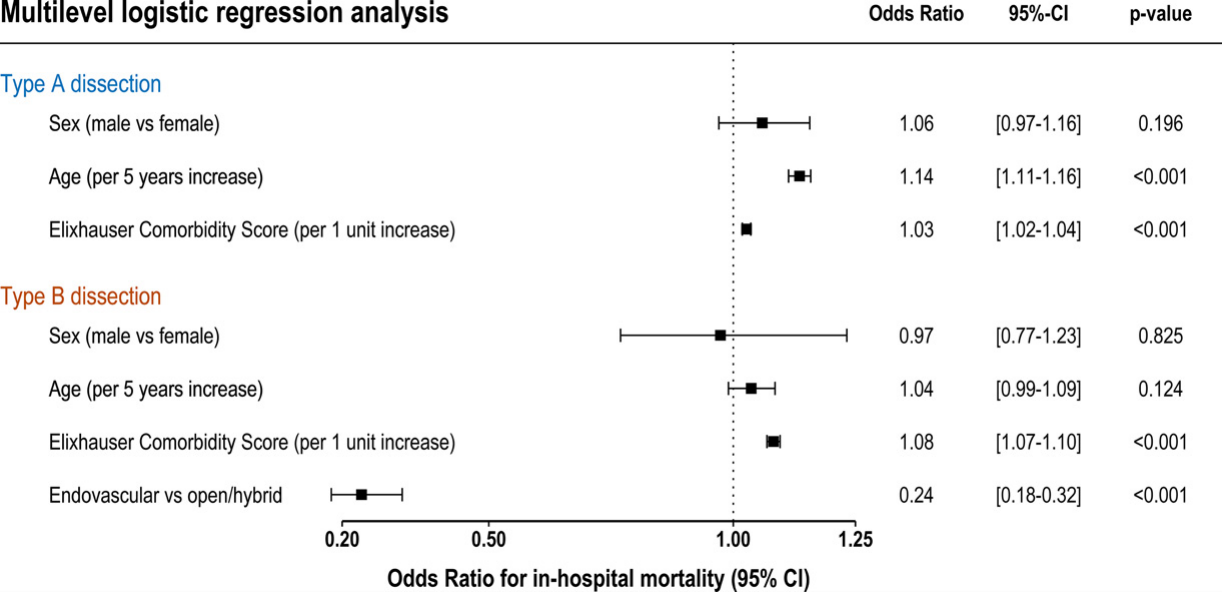
Forest plot of the multivariable analysis of factors associated with in‐hospital mortality of type A and type B aortic dissections.

The multilevel multivariable analysis revealed that age (odds ratio, 1.14; 95% CI, 1.11–1.16; *P*<0.001; per 5‐year increase) and Elixhauser Comorbidity Score (odds ratio, 1.03; 95% CI, 1.02–1.04; *P*<0.001; per 1 scoring point of the Elixhauser Comorbidity Score) were associated with higher in‐hospital mortality in TAAD. In TBAD, higher mortality was significantly associated with the Elixhauser Comorbidity Score (odds ratio, 1.08; 95% CI, 1.07–1.10; *P*<0.001). Patients treated endovascularly had a significantly lower risk of in‐hospital death (odds ratio, 0.24; 95% CI, 0.18–0.32; *P*<0.001). In neither type of AD was mortality significantly associated with sex (male versus female; Figure 7).

### Distribution of Center Volume and Volume‐Outcome Association

**Figure 8:**
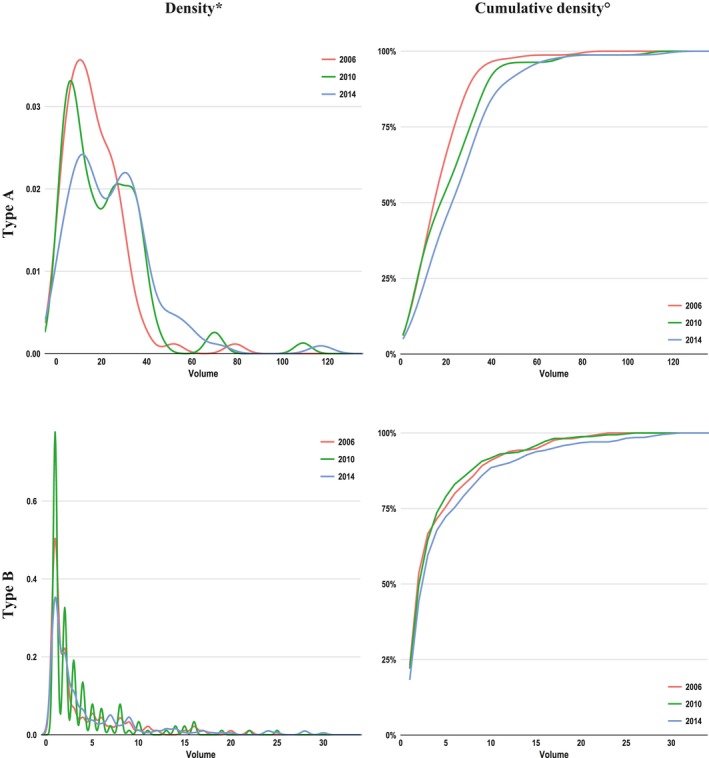
Distribution of hospital annual case volumes from 2006 (red), 2010 (green), and 2014 (blue). *As histograms giving absolute number of hospitals by volume were prohibited because of the data protection law, kernel‐density plots were used for approximation. °Cumulative density indicates the share of hospitals having an annual case volume equal to or less than indicated on the *x* axis. For example, in 2006, ≈65% of hospitals operated on ≤20 type A aortic dissection cases, whereas in 2014, this share decreased to 44%.

From 2006 to 2014, the relative number of low‐volume centers decreased (Figure 8). For example, in 2006, ≈65% of hospitals operated ≤20 TAAD cases, whereas in 2014, this share decreased to 44%. In TAAD, volume distribution shifted from a right‐skewed distribution in 2006 toward a 2‐peaked distribution comprising also higher maximum values in 2014. For volume‐outcome effects in TAAD, the in‐hospital mortality decreased with increasing annual center volumes and was 22%, 19%, and 17% in low‐, middle‐, and high‐volume centers, respectively (Table 6). In TBAD, these figures were 11%, 9%, and 7%, respectively. After adjustment for age, sex, Elixhauser Comorbidity Score, and technique of operation (the latter only for TBAD), the inverse association between higher annual case volume and lower in‐hospital mortality was statistically significant in TAAD (*P*<0.001), but not in TBAD (*P*=0.130).

**Table 6:** Volume Outcome Analyses

Type of AD	Low‐Volume Cluster^a^	Medium‐Volume Cluster^a^	High‐Volume Cluster^a^	Volume‐Outcome Effect (*P* Value)
TAAD				
Hospital volume	9 (1**–**20)	29 (21**–**53)	70 (55**–**127)	···
No. of hospitals^b^	44.8	32.4	2.8	···
No. of patients	4027	8937	1947	···
In‐hospital mortality	898 (22.3)	1694 (19.0)	321 (16.5)	0.001^a^ 0.001^c^
TBAD				
Hospital volume	2 (1**–**6)	10 (7**–**17)	22.5 (18**–**47)	···
No. of hospitals^b^	125.0	23.2	4.9	···
No. of patients	2362	2213	1047	···
In‐hospital mortality	248 (10.5)	196 (8.9)	78 (7.4)	0.003^a^ 0.130^c^

Data are given as number (percentage) or median (first‐third quartile), unless otherwise indicated. AD indicates aortic dissection; TAAD, type A AD; TBAD, type B AD.

a Hospitals were clustered into 3 clusters according to their annual center volume. For clustering, the k‐means algorithm was used. Center volume is given as median with 1% and 99% percentiles because output of minimum and maximum was prohibited by data protection law.

b Average numbers of hospitals per year.

Cochran‐Armitage test for trend (using raw proportions).

c Volume‐outcome effect using center volume as a continuous variable entered into a multilevel multivariable regression model, adjusting for age, sex, comorbidity (using Elixhauser Comorbidity Score), and type of therapy (only in TBAD).

## Discussion

Comprising 20 533 cases, this study is the largest population‐based analysis of surgically/interventionally treated ADs using a statutory nationwide database. It showed that the standardized hospital incidence was 2.0/100 000 per year for TAAD and 0.7/100 000 per year for TBAD. For both entities, the hospital incidence increased over time. Furthermore, the share of low‐volume hospitals decreased from 2006 to 2014, which may be indicative for increasing centralization of vascular services, especially for TAAD. For the latter, in contrast to TBAD, there was a significant inverse relationship between higher‐center volume and lower in‐hospital mortality. Besides center volume, this study reveals that age and comorbidity burden, but not sex, are independent risk factors for in‐hospital mortality.

### Incidence

The overall standardized hospital incidence of surgically/interventionally treated AD of 2.7/100 000 per year seems rather low when compared with previously reported incidences for AD, which were between 2.3 and 15/100 000 per year.^5^, ^6^, ^7^, ^8^, ^9^, ^10^, ^11^, ^12^ Zimmerman et al noted that for the National Inpatient Sample database, which uses deidentified administrative data similar to the diagnosis‐related group database, the risk of multiple counting of admissions is increased if conservatively treated cases are included.^14^ For these cases, multiple hospitalizations, especially in those with TBAD, are not uncommon (eg, if patients experience recurrent pain episodes). For patients who have been admitted for surgery, the risk of double counting was considered negligible, as patients usually receive major repair of the same aortic segment only once. Therefore, it might be that the hospital incidence of AD reported in previous studies, which included a large group of cases that had been treated conservatively, was too high because of multiple counting. As this study excluded all cases without procedure codes specific for TAAD/TBAD repair, the reported hospital incidence only considers surgically/interventionally treated patients. Thus, double counting is considered less likely.

Over the study period, the hospital incidence of both types of surgically/interventionally treated AD increased significantly. This was also observed in a previous study in the United States, with an increase from 2.7 to 4.1/100 000 per year (1.26/100 000 per 5 years),^6^ and predicted for the future in the United Kingdom by Howard et al because of demographic changes, with patients becoming increasingly older.^9^ Further reasons for the observed increase in the hospital incidence may be that more dissections can be diagnosed by the increasing availability of imaging techniques. In addition, the increased use of endovascular therapy also permits less invasive treatment of patients who were previously considered unfit for surgery. Another reason could be that more uncomplicated TBADs are treated endovascularly, which could be a consequence of the findings of the INSTEAD XL (Investigation of Stent Grafts in Patients with Type B Aortic Dissection) trial.^4^ The latter led to the recommendation in the European guidelines that an intervention should be considered in uncomplicated TBAD.^3^

### In‐Hospital Mortality

The in‐hospital mortality for TAAD was ≈20%; and for TBAD, ≈9%. This was within the range of smaller studies, which reported mortality rates for TAAD of 17.9% to 21.1% and for TBAD of 7.9% to 26.9%.^8^, ^14^ In both types of AD, mortality increased over the 9‐year period despite improved diagnosis and perioperative therapy. In a trend analysis, even a significant increase in TAAD was noted. One reason could be that more and more higher‐risk patients were treated, especially patients with TAAD. The latter hypothesis is underpinned by the finding that the Elixhauser Comorbidity Score increased over the years (Figure 2) and that the Elixhauser Comorbidity Score was associated with higher mortality risk. In addition, it could be shown in a comparison of survivors versus nonsurvivors for both types of AD (Tables 4 and 5) that nonsurvivors were significantly older (5 years in TAAD and 4 years in TBAD); experienced more comorbidities, especially from cardiovascular disease (chronic heart failure and cerebrovascular disease); and had more postoperative complications. No statistically significant difference in age over the years was observed (Figure 3).

Other reasons for the increase in mortality are speculative. It is known that the turn‐down rate of patients who did not receive any kind of surgical treatment in TAAD was 28% in 2000 in the International Registry of Acute Aortic Dissections,^32^ and declined to 11% in 2015.^13^ The fact that surgery was offered to patients who were previously denied could possibly be one of the reasons for the significant increase in TAAD mortality. In the case of TBAD, although an increase of the in‐hospital mortality was apparent, this was not significant. This could be because of the increased use of endovascular therapy, which was also associated with lower mortality in the multivariable analysis. Overall, the increase in mortality is contrary to the studies based on the National Inpatient Sample database^14^, ^15^ as well as the recent regional epidemiological study for the Ontario, Canada, region, which all reported a decreasing mortality in patients treated for AD.^12^ The multivariable analysis showed that the mortality of neither type of AD was associated with sex, as had also been shown in the International Registry of Acute Aortic Dissections.^33^ However, this is in contrast to infrarenal aortic surgery having a higher mortality among women.^22^

Between 2006 and 2014, the annual volume of treated patients has increased across hospitals (Figure 8). In contrast to TBAD, volume‐outcome analysis in TAAD revealed that high center volume was associated with low mortality (Table 6). This volume‐outcome effect is in line with findings in other vascular pathological conditions, such as aortic aneurysms (abdominal,^21^ thoracic,^23^ and thoracoabdominal^24^) or carotid stenosis.^34^ Overall, these empirical findings may be considered indicative for increasing centralization of TAAD and TBAD treatment and a significant volume‐outcome effect for TAAD.

Open surgery remains the most widely performed procedure in TAAD, with the proportion of hybrid procedures increasing. The proportion of endovascular treatments, including hybrid procedures, is increasing steadily in TBAD, and had already reached nearly 97% in 2014 (from 89% in 2006).

The proportion of patients with TAAD transferred to another hospital on discharge was relatively high (33.3%). It is impossible to say which nonaortic treatments, especially treatment of any procedure‐dependent complications, were performed there. Furthermore, the mortality of this cohort may be even higher, as nothing is known about whether the patients died in the next hospital. Overall, it remains difficult to determine the true incidence and mortality of AD, especially TAAD, because many patients die before hospitalization and the autopsy rate is decreasing significantly, at least in Germany.^35^, ^36^

### Limitations

This study has several limitations, which are listed below:

- The data are administrative rather than clinical; thus, coding policy could lead to biased diagnosis or procedure coding and consequently to wrong case selection (trade‐offs between scale and depth^16^). Because the data were controlled by an external institution (Medizinischer Dienst der Krankenkassen), up coding of procedures and principal diagnoses is considered minor. In addition, risk of down coding (or noncoding) of procedures is considered low because a set of documented procedures is needed for board certification and hospital remuneration in Germany. In summary, noncoding is counterbalanced by 2 separate forces and up coding by external monitoring; thus, information bias is considered low. However, underreporting of secondary diagnoses or procedures may occur if they are not relevant for reimbursement.
- Selection bias: This study only includes patients who were admitted to the hospital (or reached the hospital alive). In addition, because of the data protection law, there is no nationwide unique patient identification and, thus, readmissions could not be linked to earlier hospital episodes. To avoid double (or multiple) counting, only those patients who underwent surgery/intervention were included. Therefore, interpretation of the presented data should consider a possible selection bias.
- A statement about the exact timing of surgery after symptom onset, which would be of particular interest in TBAD (acute, subacute, or chronic), cannot be provided on the basis of the data.
- It cannot be said how many of the surgically treated TBADs were uncomplicated or chronic.
- Furthermore, it cannot be shown which patients did not receive surgery because they were considered moribund.
- Because the data only cover the hospital stay, no statements can be made about follow‐up.

## Conclusion

Comprising 20 533 cases, this study is the largest population‐based analysis of surgically/interventionally treated ADs using a statutory nationwide database. It showed that the standardized hospital incidence was 2.0/100 000 per year for TAAD and 0.7/100 000 per year for TBAD. For both entities, the hospital incidence increased over time. Furthermore, the share of low‐volume hospitals decreased from 2006 to 2014, which may be indicative for increasing centralization of vascular services, especially for TAAD. For the latter, in contrast to TBAD, there was a significant inverse relationship between higher center volume and lower in‐hospital mortality. Besides center volume, this study reveals that age and comorbidity burden, but not sex, are independent risk factors for in‐hospital mortality.

## Sources of Funding

This work was supported by the German Research Foundation and the Technical University of Munich in the framework of the Open Access Publishing Program.

## Disclosures

None.

## Supporting information


**Table S1.** Definitions of the Different Types of Aortic Dissections Based on the Combination of Codes for Diagnosis (ICD‐10‐GM; source: www.dimdi.de) as Well as for Operations and Procedures (OPS)
