# Supplementary material for: Hospital Incidence and In‐Hospital Mortality of Surgically and Interventionally Treated Aortic Dissections: Secondary Data Analysis of the Nationwide German Diagnosis‐Related Group Statistics From 2006 to 2014
Source: J Am Heart Assoc. 2019 Apr 12;8(8):e011402. doi: 10.1161/JAHA.118.011402 (PMC6507201; doi:10.1161/JAHA.118.011402)
Supplement: Supplementary file 1 — Table S1. Definitions of the Different Types of Aortic Dissections Based on the Combination of Codes for Diagnosis (ICD‐10‐GM; source: www.dimdi.de) as Well as for Operations and Procedures (OPS) [file JAH3-8-e011402-s001.pdf]

SUPPLEMENTAL MATERIAL

Table 1. Definitions of the different types of aortic dissections based on the combination of codes for diagnosis (ICD-10-GM; source: www.dimdi.de) as well as for operations and procedures (OPS).

Definition	Source	Codes
Aortic dissection type A (TAAD)	ICD-10-GM	I71.00, I71.01, I71.03, I71.04, I71.05, I71.07
	+ OPS	5-35, 5-36, 8-851, 5-384.0, 5-384.1, 5-384.2 and 5-384.b*, 5-38a-a*, 5-384.8, 5384.9*, 5-384.a in combination with 8-851.
Aortic dissection type B (TBAD)	ICD-10-GM	I71.00, I71.01, I71.02, I71.03, I71.04, I71.05, I71.06, I71.07
	+ OPS	5-384.3, 5-384.4, 5-384.5, 5-384.6, 5-384.7, 5-38a.70, 5-38a.71, 5-38a.72-7a, 5-38a.8, 5-38a.80, 5-38a.81-8b, 5-38a.0, 5-38a.1, 5-38a.b*, 5-384.c*
In detail for type A aortic dissections (TAAD)		
I71.00	ICD-10-GM	Dissection of unspecified site of aorta, without rupture
I71.01	ICD-10-GM	Dissection of thoracic aorta, without rupture
I71.03	ICD-10-GM	Dissection of thoracoabdominal aorta, without rupture
I71.04	ICD-10-GM	Dissection of unspecified site of aorta, ruptured
I71.05	ICD-10-GM	Dissection of thoracic aorta, ruptured
I71.07	ICD-10-GM	Dissection of thoracoabdominal aorta, ruptured
For "open repair" of TAAD		
5-35	OPS	Operation of heart valves and cardiac vessels
5-36	OPS	Operation of coronary arteries
8-851	OPS	Use of heart lung machine
5-384.0	OPS	Open repair: Resection and replacement (interposition) of the ascending aorta
5-384.1	OPS	Open repair: Resection and replacement (interposition) of the ascending aorta with reimplantation of coronary arteries
5-384.2	OPS	Open repair: Resection and replacement (interposition) of aortic arch
For "hybrid repair" of TAAD (OPS codes only in combination with 8-851 (use of heart lung machine)		
5-384.b*	OPS	Ascending / arch / thoracic aorta with hybrid procedure including an implantation of one (or more) stentgraft(s); code used in the years 2011-2012
5-38a-a*	OPS	hybrid procedures of the ascending / arch / thoracic aorta, with implantation of one (or more) stentgraft(s); code used in the years 2013-2014
5-384.8	OPS	Resection and replacement (interposition) of the aorta, ascending aorta, aortic arch and descending aorta with hybrid prosthesis; code used in the years 2006-2009 and 2011-2014
5384.9*	OPS	Resection and replacement (interposition) of the aorta, ascending aorta, aortic arch or thoracic aorta with hybrid prosthesis; code used in the year 2010

5-384.a	OPS	Resection and replacement (interposition) of the aorta or thoracoabdominal aorta with hybrid prosthesis; code used in the year 2010
In detail for type B aortic dissections (TBAD)		
I71.00	ICD-10-GM	Dissection of unspecified site of aorta, without rupture
I71.01	ICD-10-GM	Dissection of thoracic aorta, without rupture
I71.02	ICD-10-GM	Dissection of abdominal aorta, without rupture
I71.03	ICD-10-GM	Dissection of thoracoabdominal aorta, without rupture
I71.04	ICD-10-GM	Dissection of unspecified site of aorta, ruptured
I71.05	ICD-10-GM	Dissection of thoracic aorta, ruptured
I71.06	ICD-10-GM	Dissection of abdominal aorta, ruptured
I71.07	ICD-10-GM	Dissection of thoracoabdominal aorta, ruptured
For "open repair" of TBAD		
5-384.3	OPS	Resection and replacement (interposition) of the descending aorta
5-384.4	OPS	Resection and replacement (interposition) of the thoracoabdominal aorta
5-384.5	OPS	Resection and replacement (interposition) of the abdominal aorta, not further described
5-384.6	OPS	Resection and replacement (interposition) of the abdominal aorta, suprarenal
5-384.7	OPS	Resection and replacement (interposition) of the abdominal aorta, infrarenal
For "endovascular repair" of TBAD		
5-38a.70	OPS	Thoracic endovascular aortic repair (TEVAR) of the thoracic aorta, without fenestrations or branches
5-38a.71	OPS	TEVAR with fenestrations or branches of the thoracic aorta; code used in the years 2006-2009
5-38a.72-7a	OPS	TEVAR with fenestrations or branches of the thoracic aorta; code used in the years 2010-2014
5-38a.8	OPS	TEVAR with or without fenestrations or branches of the thoracoabdominal aorta; code used in the years 2006-2009
5-38a.80	OPS	TEVAR without fenestrations or branches of the thoracoabdominal aorta; code used in the years 2010-2014
5-38a.81-8b	OPS	TEVAR with fenestrations or branches of the thoracoabdominal aorta; code used in the years 2010-2014
5-38a.0	OPS	Endovascular aortic repair aorta, not further described
5-38a.1	OPS	Endovascular aortic repair abdominal aorta
For "hybrid repair" of TBAD		
5-38a.b*	OPS	Repair of the thoracoabdominal aorta with hybrid procedure and implantation of one or more stentgrafts; codes used 2011 and 2012
5-384.c*	OPS	Repair of the thoracoabdominal aorta with hybrid procedure and implantation of one or more stentgrafts, codes used 2013 and 2014

Characteristics and secondary diagnosis (Table 1)		
Elixhauser Comorbidity Score (ES)	Literature	Elixhauser[1], van Walraven et al.[2]
Chronic ischemic heart disease	ICD-10-GM	I25*
Chronic heart failure	ICD-10-GM	I50*
Cerebrovascular disease	ICD-10-GM	G45*, G46*, H340*, I6*
Hypertension	ES[24]	Elixhauser Item No. 6
Chronic pulmonary disease	ES[24]	Elixhauser Item No. 9
Diabetes mellitus	ES[24]	Elixhauser Item No. 10 and 11
Chronic kidney disease	ES[24]	Elixhauser Item No. 13
Cancer	ES[24]	Elixhauser Item No. 17, 18 and 19
Obesity	ES[24]	Elixhauser Item No. 22
Marfan-Syndrome	ICD-10-GM	Q87.4
Perioperative management and complications (Table 2, Table 3)		
Prolonged ventilation	-	Directly coded variable *(hours of ventilation after leaving the operating room)*
Acute/Recurrent myocardial infarction	ICD-10	I21.*, I22.*
Acute stroke	ICD-10	I63.4
Acute paraplegia (incl. spinal infarction)	ICD-10	G95.1
Acute limb ischemia	ICD-10	I74.*
Major amputation lower limb	OPS	5-864.*
Acute mesenteric infarction	ICD-10	K55.0
Resection of bowel	OPS	5-45*
Acute renal artery infarction	ICD-10	N28.0
Blood transfusion 1-5 units	OPS	8-800.c0, 8-8007f
Blood transfusion >5 units	OPS	8-800.7* without 8-800.7f<br>8-800.c* without 8-800.c0
Transfusion of thrombocytes	OPS	8-800.6*, 8-800.8*, 8-800.a*, 8-800.b*, 8-800.e*, 8-800.9*, 8-800.d*

ICD-10-GM = International Classification of disease, tenth revision, German Modification; OPS = Operation and Procedure Code, DIMIDI = German Institute of Medical Documentation and Information.ES= Elixhauser Comorbidity Score.

Supplemental References:

1. Elixhauser A, Steiner C, Harris DR, Coffey RM. Comorbidity measures for use with administrative data. *Med Care*. 1998;36:8–27.

2. van Walraven C, Austin PC, Jennings A, Quan H, Forster AJ. A modification of the Elixhauser comorbidity measures into a point system for hospital death using administrative data. *Med Care*. 2009;47:626–633.
